# Identification of a 3-Alkylpyridinium Compound from the Red Sea Sponge *Amphimedon chloros* with *In Vitro* Inhibitory Activity against the West Nile Virus NS3 Protease

**DOI:** 10.3390/molecules23061472

**Published:** 2018-06-18

**Authors:** Aubrie O’Rourke, Stephan Kremb, Brendan M. Duggan, Salim Sioud, Najeh Kharbatia, Misjudeen Raji, Abdul-Hamid Emwas, William H. Gerwick, Christian R. Voolstra

**Affiliations:** 1 Red Sea Research Center, Division of Biological and Environmental Science and Engineering (BESE), King Abdullah University of Science and Technology (KAUST), Thuwal 23955-6900, Saudi Arabia; aubrie.orourke12@gmail.com (A.O.); sk6225@nyu.edu (S.K.); 2 Scripps Institution of Oceanography and Skaggs School of Pharmacy and Pharmaceutical Sciences, University of California San Diego, 9500 Gilman Drive, La Jolla, CA 92093, USA; wgerwick@ucsd.edu; 3 King Abdullah University of Science and Technology (KAUST), Core Labs, Thuwal 23955-6900, Saudi Arabia; salim.sioud@kaust.edu.sa (S.S.); najeh.kharbatia@kaust.edu.sa (N.K.); rajimi@sheridancollege.ca (M.R.); abdelhamid.emwas@kaust.edu.sa (A.-H.E.)

**Keywords:** halitoxin, antiviral, Red Sea, bioprospecting, West Nile Virus, NS3 protease, High-Content Screening (HCS)

## Abstract

Viruses are underrepresented as targets in pharmacological screening efforts, given the difficulties of devising suitable cell-based and biochemical assays. In this study we found that a pre-fractionated organic extract of the Red Sea sponge *Amphimedon chloros* was able to inhibit the West Nile Virus NS3 protease (WNV NS3). Using liquid chromatography–mass spectrometry (LC-MS) and nuclear magnetic resonance (NMR) spectroscopy, the identity of the bioactive compound was determined as a 3-alkylpyridinium with *m*/*z* = 190.16. Diffusion Ordered Spectroscopy (DOSY) NMR and NMR relaxation rate analysis suggest that the bioactive compound forms oligomers of up to 35 kDa. We observed that at 9.4 μg/mL there was up to 40–70% inhibitory activity on WNV NS3 protease in orthogonal biochemical assays for solid phase extracts (SPE) of *A. chloros*. However, the LC-MS purified fragment was effective at inhibiting the protease up to 95% at an approximate amount of 2 µg/mL with negligible cytotoxicity to HeLa cells based on a High-Content Screening (HCS) cytological profiling strategy. To date, 3-alkylpyridinium type natural products have not been reported to show antiviral activity since the first characterization of halitoxin, or 3-alkylpyridinium, in 1978. This study provides the first account of a 3-alkylpyridinium complex that exhibits a proposed antiviral activity by inhibiting the NS3 protease. We suggest that the here-described compound can be further modified to increase its stability and tested in a cell-based assay to explore its full potential as a potential novel antiviral capable of inhibiting WNV replication.

## 1. Introduction

Viruses are underrepresented as targets in pharmacological screening efforts. This is because cell-based assays of viruses are innately complex and require biochemical assay counterscreens causing them to be time consuming and expensive. These challenges cause antiviral assays to be prioritized in the industry setting in favor of the most pressing viruses such as human immunodeficiency virus (HIV) and hepatitis C virus (HCV) [1]. West Nile fever is considered a neglected tropical disease. Its causative agent, West Nile Virus (WNV), belongs to the Flaviviridae family together with the hepatitis C virus (HCV). WNV is transmitted by *Culex* spp. mosquitoes. Symptoms of the disease include fever, headache, body aches, skin rash, swollen lymph glands and in extreme cases, encephalitis or meningitis [2]. The virus is mainly endemic to Africa, the Middle East and the area around the Mediterranean Sea [3], but remains a threat to other countries when the infected hosts, both mosquitoes and humans, travel. HCV currently has more than two dozen prospective antivirals in various clinical phases, whereas WNV has none. However, WNV is well-defined biochemically and commercial biochemical assays are available for screening purposes [4]. This allows for the rapid identification of novel inhibitors from new sources.

The replication cycle of the WNV begins as the 45–50 nm enveloped, icosahedral nucleocapsid binds to unknown receptors on the host cell. Receptor-mediated endocytosis brings the virion into the cell. The positive single-stranded 11 kb RNA genome is uncoated and can be directly translated by the host machinery into single long polyproteins in the rough endoplasmic reticulum (ER) [5]. The polyprotein contains three structural proteins (capsid, pre-membrane, and envelope) and seven non-structural (NS) proteins (NS1, NS2A, NS2B, NS3, NS4A, NS4B, NS5). The polyprotein must be cleaved by the NS3 protease in order to produce the individual proteins required for replication and particle maturation [6]. A molecule that can inhibit the NS3 protease will prohibit subsequent replication, packaging, and spread of the virus within the host organism. Viral proteases are good drug targets as exemplified by the ten clinically approved HIV protease inhibitors [7] and two approved inhibitors of the HCV NS3 protease [8]. As a result, the WNV NS3 protease is a promising target for screening efforts directed at inhibiting WNV replication [9]. The WNV NS3 protease is a serine protease with a marked homology to the four serotypes of the dengue virus (DENV) NS3 protease, where all have been described to have an active site that is relatively flat and highly exposed [6], two features that pose a problem for identifying inhibitors. As a result, the non-competitive binding site has also become a targeted domain.

A well-represented group of marine natural products with unique structures are the halitoxins. These 3-alkylpyridinium molecules have acquired a number of hyponyms since the first isolation of the parent molecule, halitoxin, from a *Haliclona* sp. sponge [10]. The first compound from *Haliclona* was investigated for its characteristic ichthyotoxicity. Accounts of the halitoxin family bioactivity since the first isolation include epidermal growth factor (EGF) receptor activation [11], histone deacetylase inhibition [12], acetylcholinesterase inhibition and selective toxicity toward non-small cell lung cancer cells [13,14], dorsal root ganglion (DRG) neuron activity [15], antifungal [16], antimycobacterial, and antimicrobial [17] activity as well as cytotoxicity [10,18]. The caveat to cytotoxicity is that 3-alkylpyridiniums can be found as cyclic or linear compounds, as monomers or as polymeric alkylpyridiniums (poly-APS), where the cytotoxicity of the molecules is both size- and dose-dependent [17]. Interestingly, macrocylic compounds in general are noted for their flexibility, high potency, selectivity, solubility, lipophilicity, membrane permeability, oral bioavailability, and metabolic stability and function as chemotherapeutics, immunosuppressants, antifungals, antiparasitics, antibacterials, and antivirals [19]. However, to date none of the various forms of a 3-alkylpyridinium natural product have been reported to display any antiviral activity.

In this study a sponge-derived natural product library was screened for the ability to inhibit the WNV NS3 protease. This led to the identification of a fraction from the Red Sea sponge, *Amphimedon chloros*, that displays the ability to inhibit the WNV NS3 protease *in vitro*. This activity is attributed to the 3-alkylpyridinium derivative present in the active fraction, which in its simplest form, consists of an alkyl group connected to C-3 of a pyridine ring. An investigation into halitoxin and the 3-alkyl- pyridinium class of compounds revealed the long-standing problem of how to ascertain the relative molecular size of these compounds. This question was addressed by employing the 2D NMR DOSY technique. The technique in combination with an algorithm reported by Evans et al. [20] provides a method by which to determine the molecular weight of a compound by its rate of diffusion. Knowing the molecular weight of the compound isolated from *A. chloros* allowed for the direct comparison of the cytotoxicity of the isolated halitoxin to cytotoxicity reports for other halitoxins of various sizes. Provided here is the first account of the antiviral potential for the halitoxin family of compounds.

## 2. Results

### 2.1. A. chloros Demonstrates Inhibition of West Nile Virus NS3 Protease

**Figure 1:**
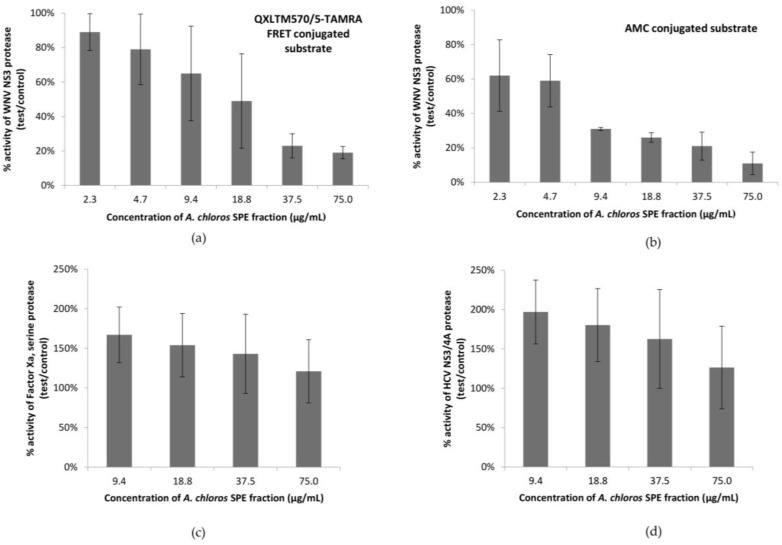
Percent activity of a series of serine proteases after treatment with SPE fraction 2 (50% IPA/H_2_O) from *A. chloros* in orthogonal biochemical assays. (**a**) Percent activity of the WNV NS3 protease in a biochemical assay with a QXLTM570/5-TAMRA FRET conjugated substrate; (**b**) Percent activity of the WNV NS3 protease in a biochemical assay with an AMC conjugated substrate; (**c**) Percent activity of the Thrombin-Factor Xa, serine protease complex in a biochemical assay with a QXL 520/5-FAM FRET conjugated substrate; (**d**) Percent activity of the HCV NS3/4A protease in a biochemical assay with a QXL 520/5-FAM FRET conjugated substrate*.* Error bars denote standard deviations.

Solid phase extracts (SPE) for three biological replicates of *A. chloros* were generated and screened for inhibitory activity against the WNV NS3 protease in two orthogonal biochemical assays. One assay used a QXLTM570/5-TAMRA FRET conjugated substrate and the second used a different, AMC-conjugated substrate to ensure that a potential hit was not a false positive resulting from the compound interacting with the fluorophore. SPE fraction 2 (50% Isopropyl Alcohol (IPA)/H_2_O) displayed the strongest inhibitory activity in all three biological replicates, and thus was further screened in a 1:2 dilution series (Figure 1a,b). The results obtained from screening six technical replicates of the three biological replicates in a serial dilution were averaged and revealed an 80–90% inhibition of the NS3 protease activity when tested at 75 μg/mL in both assays (Figure 1a,b). The same set of samples was screened for the ability to inhibit the HCV protease as well as Factor Xa associated with the serine protease, thrombin (Figure 1c,d). All replicates of fraction 2 failed to show inhibition of either of the additional targets.

### 2.2. Analytical Chemistry Reveals 3-Alkylpyridinium as the Bioactive Compound

**Figure 2:**
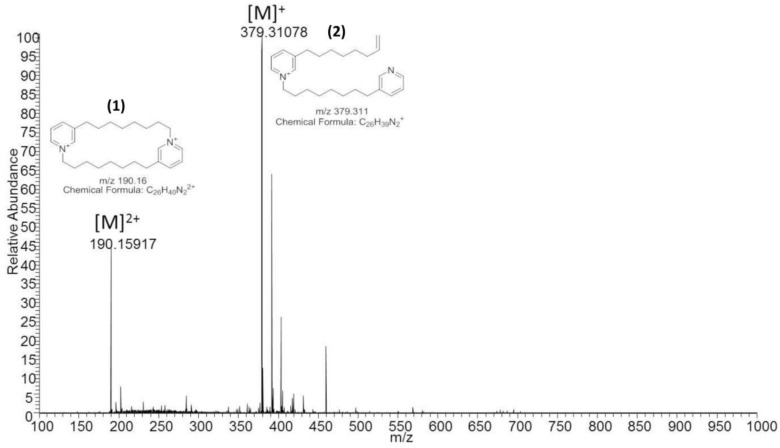
Major peaks observed during ESI(+)_MS of the active SPE fraction 2 from *A. chloros*. SPE fraction 2 of *A. chloros* shows an abundance of the linear [M − H]^+^ *m*/*z* 379.31 species, referred to as compound **2**, as well as the cyclic [M]^2+^ doubly charged species at *m*/*z* 190.16, referred to as compound **1**.

The LC-MS spectrum of the active SPE fraction 2 of *A. chloros* showed two major species; one with *m*/*z* 190.16, henceforth referred to as compound **1**, and the other with *m*/*z* 379.31, henceforth referred to as compound **2** (Figure 2). A compound with the same MS signature has been reported previously [11] where the species with *m*/*z* 190.16 was proposed to be a doubly charged, cyclic, alkylpyridinium salt (Figure 2, compound **1**) and the *m*/*z* 379.31 species, a singly charged ion with a terminal olefin formed by gas phase cleavage (Figure 2, compound **2**). Our mass accuracy of <1 ppm is consistent with these structures, and the observation of these two ions from a single chromatographic peak (Figure S1) supports their derivation from a single molecule.

Analysis of NMR spectra of the active SPE fraction 2 identified a molecular fragment with ^1^H- and ^13^C-NMR chemical shifts consistent with the cyclic molecule of compound **1** and very similar to the ^1^H-NMR chemical shifts reported previously [11]. The NMR-defined fragment matches only half of compound **1**, but this is to be expected as compound **1** is symmetrical and the methylenes in the center of the alkyl chain will be overlapping and difficult to resolve. Interestingly, the peaks of compound **1** in the 1D ^1^H-NMR spectrum (Figure S5) were broader than the peaks of the solvent (methanol-*d*_4_, δ_H_ 3.31 ppm), suggesting a larger molecular weight for the sample than for the solvent. Furthermore, the NOESY spectrum (Figure S10) showed negative peaks for the aromatic resonances, suggesting a large molecular weight, and positive peaks for the aliphatic resonances, indicative of a small molecular weight. To determine if the negative peaks were due to chemical exchange a ROESY spectrum (Figure S11) was recorded. The ROESY showed only positive peaks, thereby eliminating the possibility that chemical exchange produced the negative peaks in the NOESY. Observation of positive and negative peaks in the NOESY suggests that different parts of the molecule exhibit different rates of motion, or that the molecule is in dynamic exchange between different oligomeric states.

**Figure 3:**
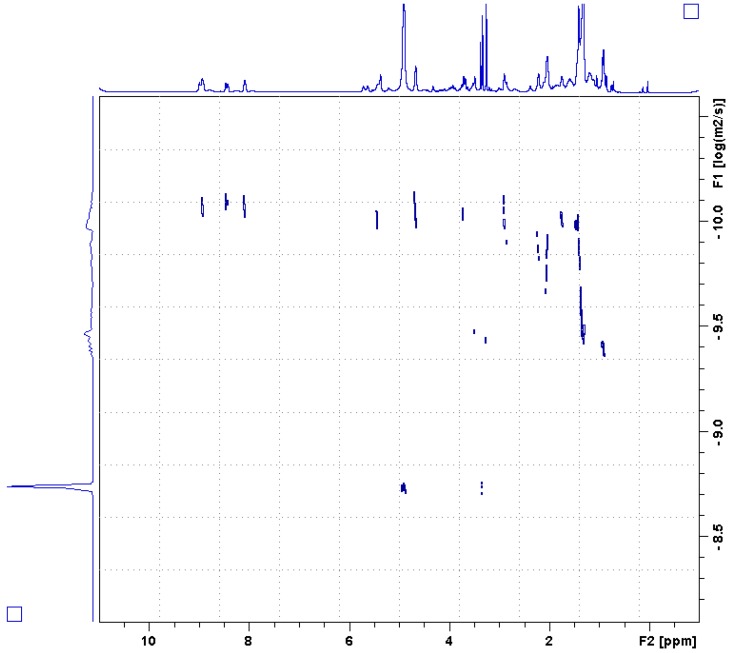
2D DOSY spectrum of active SPE fraction 2. The horizontal axis corresponds to the ^1^H spectrum, while the vertical axis corresponds to the logarithm of the translational diffusion rate, D. Three different species were resolved, the solvent methanol (log(D) = −8.75 m^2^/s), a lipid like compound (log(D) = −9.45 m^2^/s), and the cyclic, alkylpyridinium salt compound **1** (log(D) = −10.05 m^2^/s).

The prior report of compound **1** was unable to determine if the molecule was a polymer or a large oligomer [11]. Mass spectrometric analysis could conceivably dissociate an oligomer or fragment a polymer into its monomeric subunits, and thus not describe the true size of the molecule. To better characterize the size of compound **1**, we resorted to NMR techniques that report on the state of the molecule in solution. Firstly, a 2D Diffusion Ordered Spectroscopy (DOSY) spectrum was recorded. The DOSY experiment resolves compounds by their translational diffusion, which can be used to estimate molecular weight. The DOSY spectrum (Figure 3) resolved the solvent methanol (log(D) = −8.75 m^2^/s), a lipid-like compound (log(D) = −9.45 m^2^/s) and compound **1** (log(D) = −10.05 m^2^/s). Converting the diffusion constants to molecular weights using a relationship calibrated with a collection of small molecules [18] gave an estimated molecular weight for compound **1** of 35,000. This surprisingly large value prompted us to find another means of estimating the molecular weight.

As a second method to estimate molecular weight of compound **1** we measured ^13^C T_1_ and T_2_ relaxation rates, used these to calculate the rotational diffusion time using known relationships [21], and then estimated the molecular weight using an empirical relationship derived from isotopically labeled proteins [22]. Relaxation rates were obtained only for the aromatic pyridinium resonances as methyl and methylene resonances introduce different relaxation pathways that complicate the analysis [23]. The ^13^C T_1_ and T_2_ relaxation rates of the aromatic resonance of compound **1** are shown in Table S3. Converting the relaxation rates to a rotational diffusion time gave a value of 1.08 ns, which corresponds to a molecular weight of approximately 700.

The discrepancy in molecular weight estimates is likely due to a dynamic equilibrium between single molecules and large oligomers. In a self-associating system with a range of sizes the DOSY experiment will emphasize the large oligomers, as the signals from the slower moving, large oligmers will be less attenuated than those from the smaller, faster moving oligomers. In the relaxation rate analysis the opposite is true. Signals from the slow moving, large oligomers will be attenuated more rapidly than those from the faster, smaller oligomers. Thus, the molecular weights estimated from the two different methods are likely to be upper and lower bounds on the actual size of the oligomer. We note that compound **1** was originally proposed to exist as a large oligomer or polymer [11].

**Figure 4:**
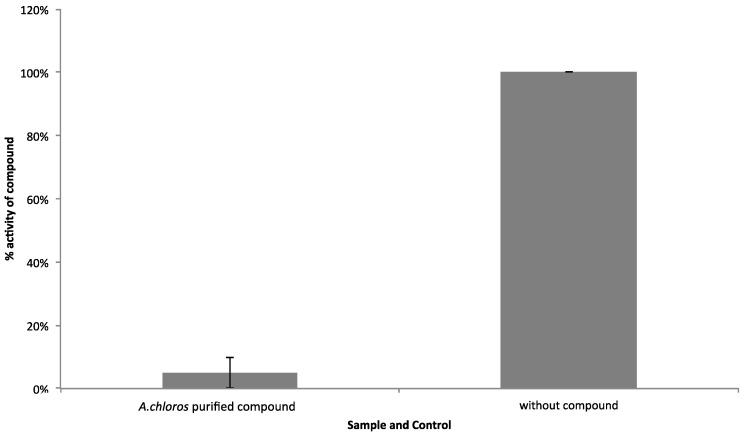
Effect of the purified 3-alkylpyridinium compound **1** from *A. chloros* on WNV NS3 protease activity. Error bars denote standard deviations.

In order to confirm that compound **1** was responsible for the WNV NS3 inhibitory activity and not the lipid contaminant identified in the DOSY, a fraction was collected (Table S1) from the region of the chromatogram (Figure S3) where compound **1** was present in the greatest relative abundance and was tested again for WNV NS3 protease inhibition (Figure 4). From this re-evaluation we could confirm with high certainty that the active compound is compound **1**, which inhibits the NS3 protease up to 95% when screened at a concentration of 2 µg/mL.

### 2.3. Cytological Profiling Reveals the Bioactive 3-Alkylpyridinium Salt as Negligibly Cytotoxic

**Figure 5:**
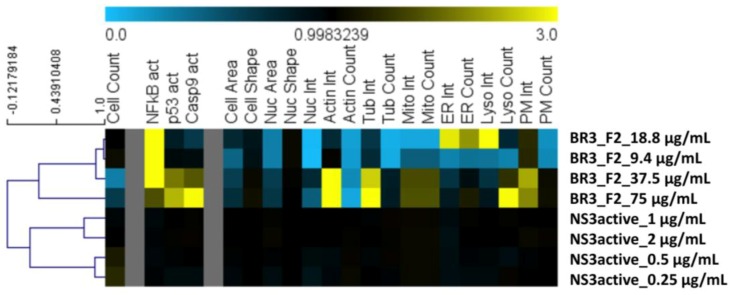
Cytolological profiles composed of 21 core features of the two parent fraction dilutions and dilutions of compound **1**. Colors indicate positive (yellow) or negative (blue) deviation from the mean of untreated control cells (value = 1). Full cytological profiles were used for clustering (left). The dendrogram depicts distances between individual cytological profiles based on Pearson correlation with average linkage for four dilutions of the parent SPE fraction (BR3_SPEfraction2_concentration) and four dilutions of the NS3 active fraction isolating compound **1** (NS3acitve_concentraion).

In order to compare cellular toxicity of the full SPE fraction 2 (Figure 1 a,b) and the purified compound (Figure 4), we screened four dilutions (75 μg/mL–9.4 μg/mL) of one of the biological replicates (BR3) of the full SPE fraction (Figure 1) and four dilutions (2 μg/mL–0.25 μg/mL) of its resulting NS3 active *m*/*z* 190.16 LC-MS focused fraction (Figure 4) on HeLa cells (Figure 5). We used a High-Content Screening (HCS) cytological profiling strategy that captures a broad panel of cellular markers to gain insight into the induced toxicity on multiple levels [24]. Compound toxicity is mostly a combination of various mechanisms, and thus, a unidimensional experimental approach is unlikely to capture the complexity of the underlying processes. We observed that the overall toxicity profile is significantly reduced for the focused fraction dilution series in comparison to the dilution series for the parent SPE fraction. The parent SPE fraction simultaneously affects a multitude of cellular processes including a marked cell loss at higher concentrations as well as strong activation of NFkB, p53 and caspase 9. In contrast, the focused fraction shows almost no effect on any of the cellular markers. The overall concentration of the focused fraction is less than the parent SPE fraction (approximately 2 μg/mL), yet NS3 activity is enhanced, suggesting that the focused fraction is not only a potent inhibitor of the WNV NS3 protease, but also exhibits negligible cytotoxicity to HeLa cells.

## 3. Discussion

The halitoxins, presumed polymers of 3-alkylpyridinium fragments, are a group of structurally diverse natural products that have mainly been isolated from marine sponges. These molecules can be found as cyclic or linear compounds and as monomeric or as polymeric alkylpyridiniums (poly-APS). They were first characterized in 1978 [10] for their characteristic toxicity to fish, and since then, a diversity of biological activities has been observed [11,12,15,16,17,18]. In this study, we have identified and characterized a 3-alkylpyridinium compound (compound **1**), which shows inhibitory activity against the WNV NS3 protease. Davies-Coleman et al. [11] synthesized the cyclic alkylpyridinium salt and found that on thin-layer chromatography (TLC) it ran much faster than the natural product they had isolated, leading them to conclude that the natural product was an oligomer or polymer. To accurately assess the molecular size of our active compound, we used DOSY and relaxation rate analysis. Our results suggest that the molecule is not a polymer but rather an oligomer, or assembly of up to 90 molecules. Notably, alkylpyridinum salts are used as surfactants and can form micelles with as few as 10 or as many as 100 molecules [25].

In our most sensitive assay, using an AMC conjugated substrate, the SPE fraction 2 that contained compound **1** showed 90% inhibition of the WNV NS3 protease at 75 μg/mL. The same set of samples was screened for the ability to inhibit the HCV protease, as well as Factor Xa (Figure 1c,d), a serine protease which leads to blood clot formation by converting prothrombin to thrombin. All replicates of fraction 2 failed to show inhibition of either of the additional serine protease targets suggesting specificity for the WNV NS3 serine protease. Instead, in both the HCV and Factors Xa assays there was an enhancement of the protease activity on the substrate, which suggests that the active compound could be increasing the activity of these proteases, possibly through a surfactant effect [26,27,28]. Like other surfactants, compound **1** most likely exhibits a critical micelle concentration (CMC) where the molecule shows an abrupt change in solution state according to temperature and electrolyte concentration. Additional serine protease targets should be screened to determine an exclusive specificity of the active compound for the WNV NS3 protease.

To gain insight into toxicity of compound **1**, we employed a High-Content Screening (HCS) cytological profiling strategy on HeLa cells assaying SPE parent fraction dilutions and dilutions of compound **1** (Figure 5). The parent fraction that contains compound **1** showed cytotoxicity for the four dilutions of 75 μg/mL to 9.4 μg/mL when screened using the HCS platform that assesses a number of cellular features commonly affected by toxic compounds (Figure 5). After the LC-MS flow-through focused on the time point where compound **1** is present in the greatest relative abundance (Figure S1), and testing again in the WNV NS3 assay with an AMC conjugated substrate, we observed that the compound **1** focused fraction, screened at 2 μg/mL, is most likely responsible for the observed WNV NS3 inhibition (Figure 4). This same focused sample was then screened on the HCS platform and no longer showed the toxicity profile associated with the SPE parent fraction (Figure 5). Our HCS results show that the fraction collected from LC-MS is nontoxic at concentrations below 9.4 µg/mL (screened at approximately 2 μg/mL), whereas, the parent fraction is toxic (Figure 5). This is in accordance with previous reports relating compound size and treatment amount to toxicity for the halitoxin family [15,17,29]. Interestingly, the parent fraction strongly affects a variety of cellular processes, even at concentrations where no reductions in cell numbers were observed, including the induction of several well-known markers of toxicity, including activation of NFkB, p53, caspase 9 as well as effects on mitochondria, ER, and the cytoskeleton, whereas the focused fraction does not. In addition, at the higher concentrations of the parent fraction with markedly reduced cell counts, we observed an increased intensity of the membrane marker which might indicate membrane-targeting activity of the active compound. The observed reduction in toxicity of the focused fraction could be due to the reduced concentration of the NS3 protease-inhibiting material; or this concentration may not be achieving the critical micelle concentration and thereby generating a large enough oligomer capable of permeating cell membranes. Future work to evaluate this compound for its potential as a WNV antiviral would require a cell-based assay to determine compound efficacy in addition to X-ray crystallography work to fully understand the mechanism of binding for this compound to the WNV NS3 protease.

## 4. Materials and Methods 

### 4.1. A. chloros Sponge Collection

The *Amphimedon chloros* specimens were collected using gardening shears from Inner Fsar reef (22°14′37.61″ N; 39°00′28.03″ E) off the coast of KAUST (Red Sea, Saudi Arabia) at 12 meters depth using SCUBA. The samples were briefly rinsed with PBS, wrapped in foil and placed on ice, then frozen to −80 °C until processing.

### 4.2. A. chloros Sponge Extraction

Small sponge specimens of 4–10 grams in weight were ground using a mortar and pestle, and then extracted with 15 mL of methanol overnight at 4 °C. The following day, the methanol extract was dried onto 150 mg of Diaion HP20SS beads in a CentriVap complete vacuum concentrator (Labconco, Kansas City, MO, USA) on low, the beads were loaded into a 25 mL Flash Cartridge (Sorbtech, Norcross, GA, USA) with 0.795 diameter Frits (Sorbtech), desalted with deionized water (15 mL, FW1, FW2), and then eluted with 15 mL of solvent in the following series: 25% IPA/H_2_O, 50% IPA/H_2_O, 75% IPA/H_2_O, 100% MeOH [27].

### 4.3. Liquid Chromatography-Mass Spectrometry (LC-MS) of A. chloros SPE Fraction

Liquid chromatography-mass spectrometry (LC-MS) was carried out on the Thermo LTQ Orbitrap instrument (Thermo Fisher Scientific, Waltham, MA, USA) in positive mode using electrospray ionization. The 10 μL of solid phase extracted (SPE) sponge material was separated using a ZORBAX Eclipse XDB-C18 LC column (Agilent Technologies, Santa Clara, CA, USA), 4.6 mm, 150 mm, 5 µm with the gradient displayed in Table S1 and a flow rate of 0.800 mL/min. 10 μL of solid phase extract was also directly injected and the desired compound was collected at a specific run time to obtain a concentrated sample of *m*/*z* 190.16 (Figure 2, compound **1**).

### 4.4. Nuclear Magnetic Resonance (NMR) of A. chloros 3-Alkyl Pyridinium

The isolated 3-alkylpyridinium compound (12 mg) dissolved in methanol-d_4_ was used to collect NMR spectra at 298 K on Avance III 600 MHz spectrometer (Bruker, Billerica, MA, USA). Standard Bruker pulse sequences were employed. Spectra were referenced to the residual methanol solvent resonance (3.31 ppm for ^1^H and 49.0 for ^13^C). The HMBC was optimized for 8 Hz couplings, the NOESY used a 500 ms mixing time, and the ROESY used a 300 ms mixing time. The DOSY experimental data were obtained using bipolar gradient pulse pairs and two spoil gradients (*ledbpgp2s* in the standard Bruker pulse sequence library). A smoothed square shape of 2.4 ms duration was used for the bipolar gradients; its strength was increased linearly from 2% to 95% of the maximum gradient strength of 53 G/cm, acquiring 32 steps of gradient levels. A gradient recovery delay of 2 ms was used and the diffusion time was set to 300 ms (D20 of 300 ms and a P30 of 2400 µs). Each spectrum collected 128 transients with a 5 s recycle delay for a total experimental time of 7.5 h. The spectrum was processed and analyzed using TopSpin 2.1 (Bruker). ^13^C relaxation rate spectra were measured in an interleaved fashion. For T_1_s 13 delays were used (10, 20, 30, 40, 50, 70, 100, 150, 200, 300, 500, 700 and 1000 ms). For the T_2_s 11 delays were used (22.4, 44.8, 67.2, 89.6, 112.0, 134.0, 179.0, 246.0, 291.0, 381.0 and 493.0 ms). Spectra were processed with the software NMRpipe [22] and rates were extracted using the software Sparky [23].

### 4.5. West Nile Virus (WNV) NS3 Protease Inhibition Assay

The West Nile Virus NS3 protease inhibition assay was carried out using the commercial kit SensoLyte®) 440 West Nile Virus Protease Assay Kit (AnaSpec, San Jose, CA, USA). The protease is a truncated form of West Nile NS3 protease (residues 1–186). Connected to it is NS2B cofactor (residues 49–96) by the linker sequence, GGGGSGGGG. Protease activity was assessed by its ability to cleave the fluorogenic peptide Pyr-RTKR-AMC, and the subsequent production of free AMC (7-amino-4-methylcoumarin) fluorophores monitored at 354 nm excitation wavelength and 442 nm emission wavelength. As well, a second orthogonal substrate cleavage experiment was carried out, which used a FRET conjugated substrate. In this assay, West Nile Virus NS3 protease inhibition was assayed using the commercial kit SensoLyte® 570 West Nile Virus Protease Assay Kit (AnaSpec). Here, WNV protease inhibitors are assessed by their ability to inhibit the cleavage of the QXLTM570/5-TAMRA FRET substrate by the protease. Upon cleavage into two separate fragments by the WNV NS3 protease, the fluorescence of 5-TAMRA can be monitored at excitation/emission = 540 nm/575 nm. All extracts and controls were performed with three replicates in a 384-well plate format, each with a total reaction mixture of 33 μL. To begin, the test extracts and protease solution were incubated at 37 °C for 10 min before the addition of the pre-heated substrate. After substrate addition and gentle mixing the reaction was incubated at 37 °C for one h. The fluorophore was detected with the use of a SpectraMax® Paradigm® Multi-mode Microplate Detection Platform (Molecular Devices, Sunnyvale, CA, USA) by scanning at the wavelength associated with the respective assay.

### 4.6. HCV NS3/4A Protease Inhibition Assay

The HCV NS3/4A protease inhibition assay was carried out using the commercial SensoLyte® 520 HCV Protease Assay Kit (AnaSpec). The HCV NS3/4A protease is a 217 amino acid fusion protein (22.7 kDa) with NS4A co-factor fused to the N-terminus of NS3 protease domain. HCV NS3/4A protease activity was assessed by its ability to cleave the fluorogenic FRET peptide, and the subsequent unquenching by QXL 520 quencher of the 5-FAM fluorophore, which emits fluorescence. All extracts and controls were performed with three replicates in a 384-well plate format, each with a total reaction mixture of 18 μL. To begin, the test extracts and protease solution were incubated at room temperature for 10 min before the addition of the substrate. After substrate addition and gentle mixing the reaction was incubated at room temperature for one hour. The fluorophore was detected with the use of a SpectraMax® Paradigm® Multi-mode Microplate Detection Platform (Molecular Devices) by scanning at 490 nm excitation wavelength and 520 nm emission wavelength.

### 4.7. Thrombin Serine Protease Inhibition Assay

The Factor Xa inhibition assay was carried out using a commercial SensoLyte® 520 Factor Xa Assay Kit (AnaSpec). The Factor Xa activity was assessed by its ability to cleave the fluorogenic FRET peptide and the subsequent unquenching by QXL 520 quencher of the 5-FAM fluorophore, which emits fluorescence. All extracts and controls were performed with three replicates in a 384-well plate format, each with a total reaction mixture of 12.5 μL. To begin, the test extracts and Thrombin/Factor Xa solution were incubated for five minutes then the substrate was added. After substrate addition and gentle mixing the reaction was incubated at room temperature for one hour. The fluorophore was detected with the use of a SpectraMax® Paradigm® Multi-mode Microplate Detection Platform (Molecular Devices) by scanning at 490 nm excitation wavelength and 520 nm emission wavelength.

### 4.8. Cytological Profiling by High-Content Screening (HCS)

Cytological profiling was performed as recently described [24,30]. Briefly, HeLa cells were tested in black µClear CELLSTAR 384-well plates (Greiner Bio-One, Kremsmünster, Austria) at an approximate density of 2000 cells per well in a volume of 25 µL of cell culture medium with re-dissolved fractions in 4 replicates. 24 h after treatment, four different cell-staining protocols (panels) were used to stain for 10 cellular targets (cell count/cell loss; cell cycle; whole cell morphology; nuclear morphology; actin; tubulin; mitochondria; lysosomes; endoplasmic reticulum; plasma membrane; activation of NFkB, p53 and caspase 9). For High-Content Analysis, the CellomicsArrayScan VTI (Thermo Fisher Scientific, Waltham, MA, USA) platform equipped with a 10x objective (Zeiss Plan Neofluar, NA 0.3, ZEISS, Oberkochen, Germany) was used. Images were analyzed using the Compartmental Analysis Bio Application software (Cellomics, Thermo Fisher Scientific). At least 500 valid objects were analyzed per well. Cell cycle analysis and analysis of cell loss were accomplished by using the Cell Cycle Bio Application (Cellomics) using a minimum of 2000 valid objects.

## Supplementary Materials

The following are available online. Figure S1. Mass spectra of ions from SPE fraction 2 active against VNV NS3; Figure S2. Extracted LC-MS chromatrograms; Figure S3. LC-MS direct injection collection of concentrated m/z 379 NS3 active compound; Table S1. LC-MS gradient used to isolate and characterize the bioactive compound of *A. chloros* SPE fraction 2; Figure S4. Fragment of compound **1** defined by NMR; Table S2. Chemical shift assignments of compound **1**; Figure S5. 1D ^1^H NMR spectrum of active SPE fraction 2; Table S3. ^13^C T_1_ and T_2_ relaxation rates; Figure S6. ^1^H-^1^H DQF-COSY spectrum of compound **1**; Figure S7. ^1^H-^13^C HSQC spectrum of compound **1**; Figure S8. ^1^H-^13^C H2BC spectrum of compound **1**; Figure S9. ^1^H-^13^C HMBC spectrum of compound **1**; Figure S10. ^1^H-^1^H NOESY spectrum of compound **1**; Figure S11. ^1^H-^1^H ROESY spectrum of compound **1**.
