# Supplementary material for: Identification of a 3-Alkylpyridinium Compound from the Red Sea Sponge Amphimedon chloros with In Vitro Inhibitory Activity against the West Nile Virus NS3 Protease
Source: Molecules. 2018 Jun 18;23(6):1472. doi: 10.3390/molecules23061472 (PMC6099703; doi:10.3390/molecules23061472)
Supplement: Supplementary file 1 [file molecules-23-01472-s001.pdf]

Supplementary Information

Identification of a 3-Alkylpyridinium Compound from the Red Sea Sponge *Amphimedonchloros* with *In Vitro* Inhibitory Activity Against the West Nile Virus NS3 Protease

Aubrie O'Rourke [1], Stephan Kremb [1], Brendan M. Duggan [2,*], Salim Sioud [3], Najeh Kharbatia [3], Misjudeen Raji [3], Abdul-Hamid Emwas [3], William H. Gerwick [2] and Christian R. Voolstra [1,*]

1 Red Sea Research Center, Division of Biological and Environmental Science and Engineering (BESE), King Abdullah University of Science and Technology (KAUST), Thuwal 23955-6900, Saudi Arabia; aubrie.orourke12@gmail.com (A.O.); sk6225@nyu.edu (S.K.)

2 Scripps Institution of Oceanography and Skaggs School of Pharmacy and Pharmaceutical Sciences, University of California San Diego, 9500 Gilman Drive, La Jolla, CA 92093, USA; wgerwick@ucsd.edu

3 King Abdullah University of Science and Technology (KAUST), Core Labs, Thuwal, 23955-6900, Saudi Arabia; salim.sioud@kaust.edu.sa (S.S.); najeh.kharbatia@kaust.edu.sa (N.K.); rajimi@sheridancollege.ca (M.R.); abdelhamid.emwas@kaust.edu.sa (A.-H.E.)

* Correspondence: christian.voolstra@kaust.edu.sa (C.R.V:); Tel./Fax: +966-12-808-2377; bmduggan@ucsd.edu (B.M.D.); Tel.: 858-534-8763

Figure S1. Mass spectra of ions from SPE fraction 2 active against WNV NS3. 2

Figure S2. Extracted LC-MS chromatograms. 3

Figure S3. LC-MS direct injection collection of concentrated *m/z* 379 NS3 active compound. 4

Table S1. LC-MS gradient used to isolate and characterize the bioactive compound of *A. chloros* SPE fraction 2. 5

Figure S4. Fragment of compound **1** defined by NMR 6

Table S2. Chemical shift assignments of compound **1**. 7

Figure S5. 1D ^{1}H-NMR spectrum of active SPE fraction 2 8

Table S3. ^{13}C T_1 and T_2 relaxation rates. 9

Figure S6. ^{1}H-^{1}H DQF-COSY spectrum of compound **1** 10

Figure S7. ^{1}H-^{13}C HSQC spectrum of compound **1** 11

Figure S8. ^{1}H-^{13}C H2BC spectrum of compound **1** 12

Figure S9. ^{1}H-^{13}C HMBC spectrum of compound **1** 13

Figure S10. ^{1}H-^{1}H NOESY spectrum of compound **1** 14

Figure S11. ^{1}H-^{1}H ROESY spectrum of compound **1** 15

Figure S1. Mass spectra of ions from SPE fraction 2 active against WNV NS3.

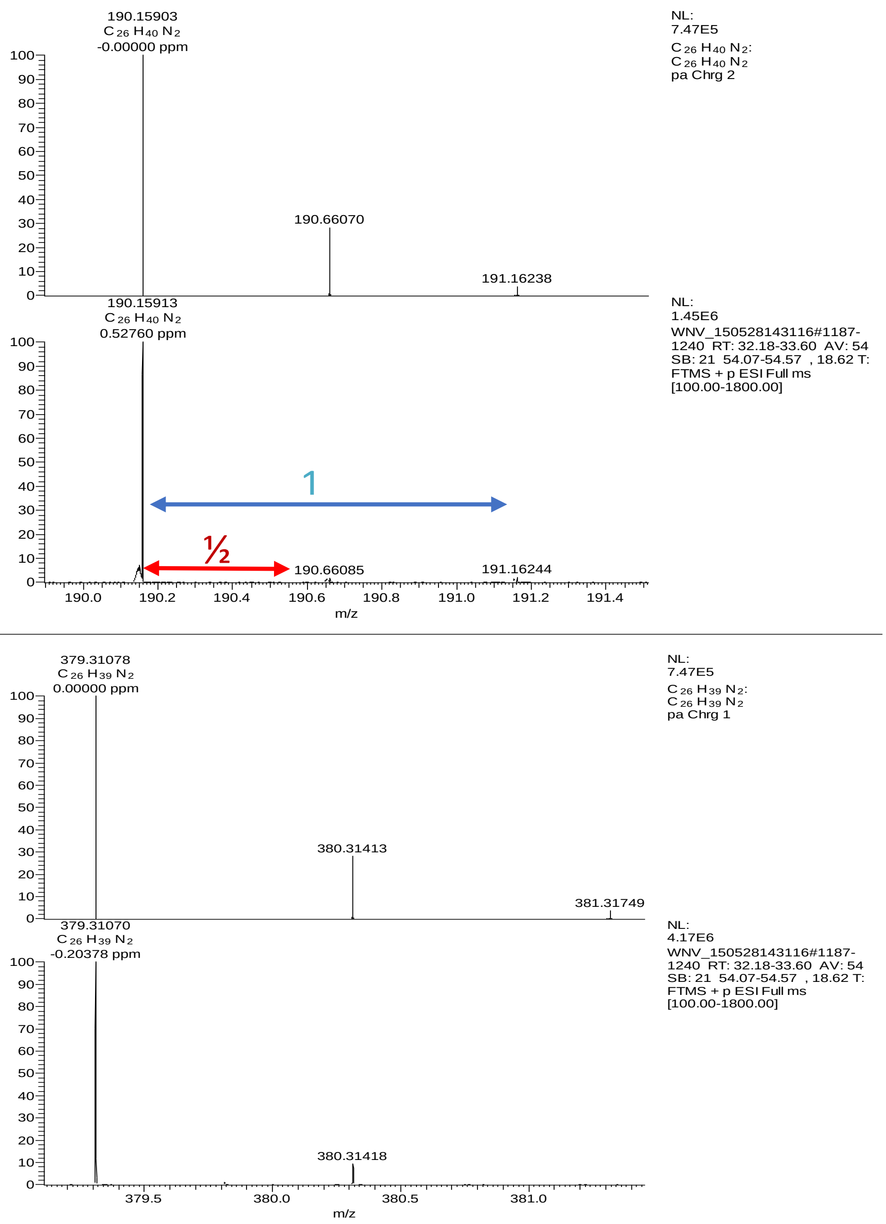

Figure S2. Extracted LC-MS chromatograms of the ions at *m*/*z* 190.15 (top, black) and at *m*/*z* 379.31 (bottom, red).

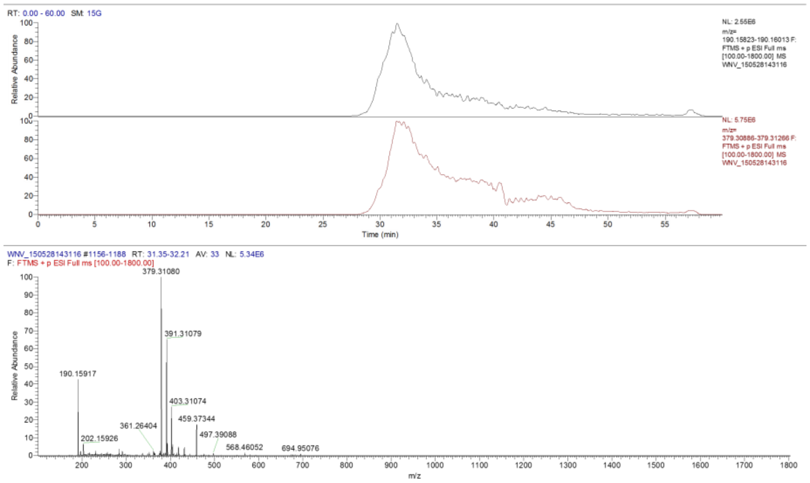

Figure S3. LC-MS direct injection collection of concentrated *m/z* 379 NS3 active compound. (a) Chromatogram showing collection time between 0.26 min and 0.33 min in the run time. (b) Display of the relative abundance of the compound present in the isolated concentrate.

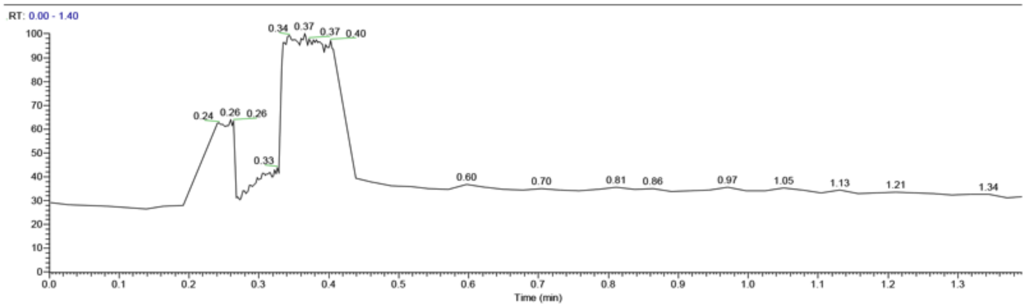

(a)

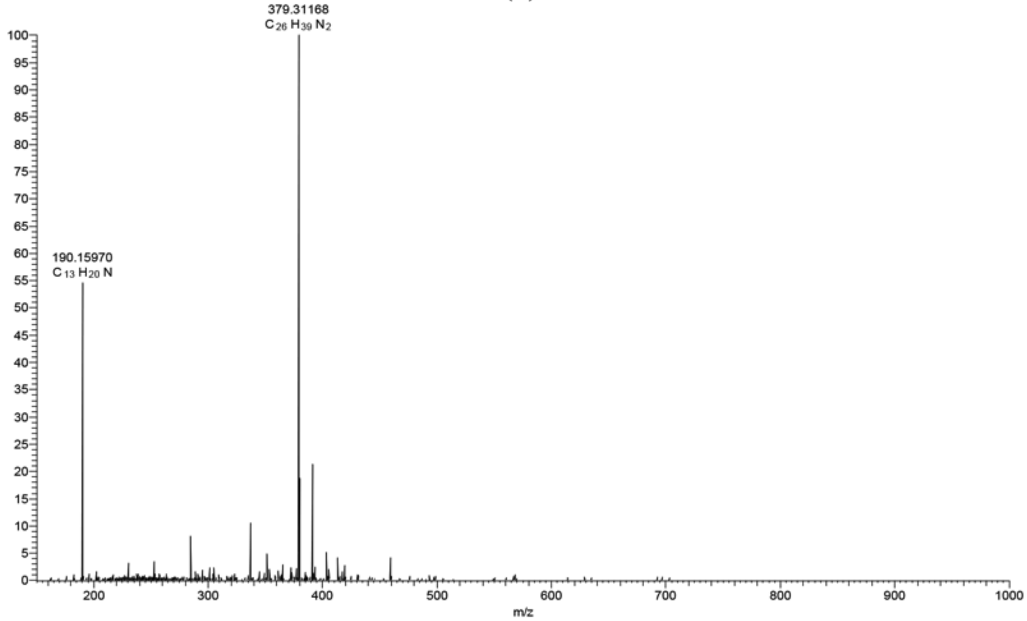

(b)

Table S1. LC-MS gradient used to isolate and characterize the bioactive compound of *A.chloros* SPE fraction 2.

Time	% H_2O	% MeOH	Formic Acid in Each Solvent
0 min	90	10	0.10%
5 min	90	10	0.10%
40 min	10	90	0.10%
50 min	10	90	0.10%
55 min	90	10	0.10%
60 min	90	10	0.10%

Figure S4. Fragment of compound **1** defined by NMR

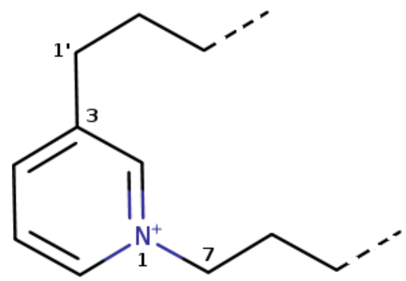

Table S2. Chemical shift assignments of compound **1.**

Position	Davies-Coleman ^{1}H (ppm)	^{1}H (ppm)	^{13}C (ppm)	COSY	H2BC	HMBC
2	8.95	8.95	144.0			3,4,6,7
3	-	-	144.4			
4	8.45	8.43	145.0	5	5	2,5,6,1'
5	8.01	8.08	127.9	4,6	4,6	2,6
6	8.84	8.89	142.3	5		2,4,5,7
7	4.60	4.64	61.5	8	8	2,6,8,9
8	2.20	2.04	31.1	7,9	7,9	7,9
9	1.41	1.40	25.7	8	8	7,8
1'	2.87	2.90	32.2	2'	2'	2,3,4,2',3'
2'	1.73	1.74	30.1	1',3'	1'	3,1',3'
3'	1.41	1.40	28.6	2'	2'	2'

Data recorded in methanol-d_4 at 298 K and 600 MHz (^{1}H) and 150 MHz (^{13}C).
The column labelled “Davies-Coleman ^{1}H (ppm)” are the chemical shifts reported in [4].
COSY correlations are to the indicated hydrogen atom.
H2BC and HMBC correlations are to the indicated carbon atom.

Figure S5. 1D ^{1}H-NMR spectrum of active SPE fraction 2 (methanol-d_4 recorded at 600 MHz and 298 K. Note the solvent peaks at δ_H 3.31 ppm are much sharper than the sample peaks).

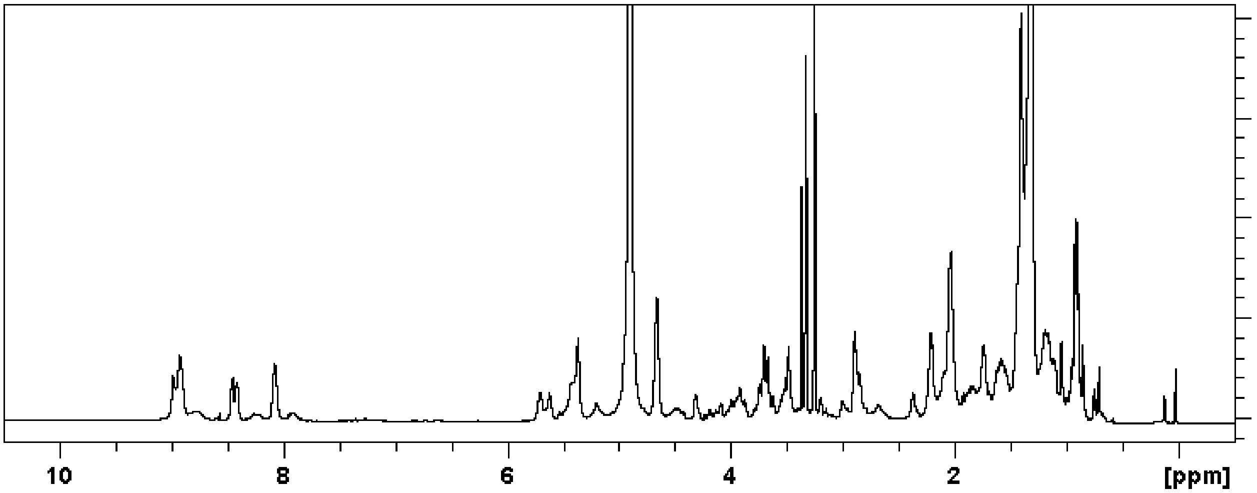

Table S3. ^{13}C T_1 and T_2 relaxation rates.

Position	T_1 (ms)	T_2 (ms)
2	442.2 ± 22.3	273.2 ± 59.5
4	325.8 ± 16.0	159.8 ± 22.5
5	441.5 ± 6.1	176.4 ± 28.3
6	369.0 ± 6.7	219.5 ± 7.3

Relaxation rates were obtained by fitting a two parameter exponential decay to peak heights. Uncertainties were estimated by refitting five times with additional Gaussian distributed noise.

Figure S6. ^{1}H-^{1}H DQF-COSY spectrum of compound **1**

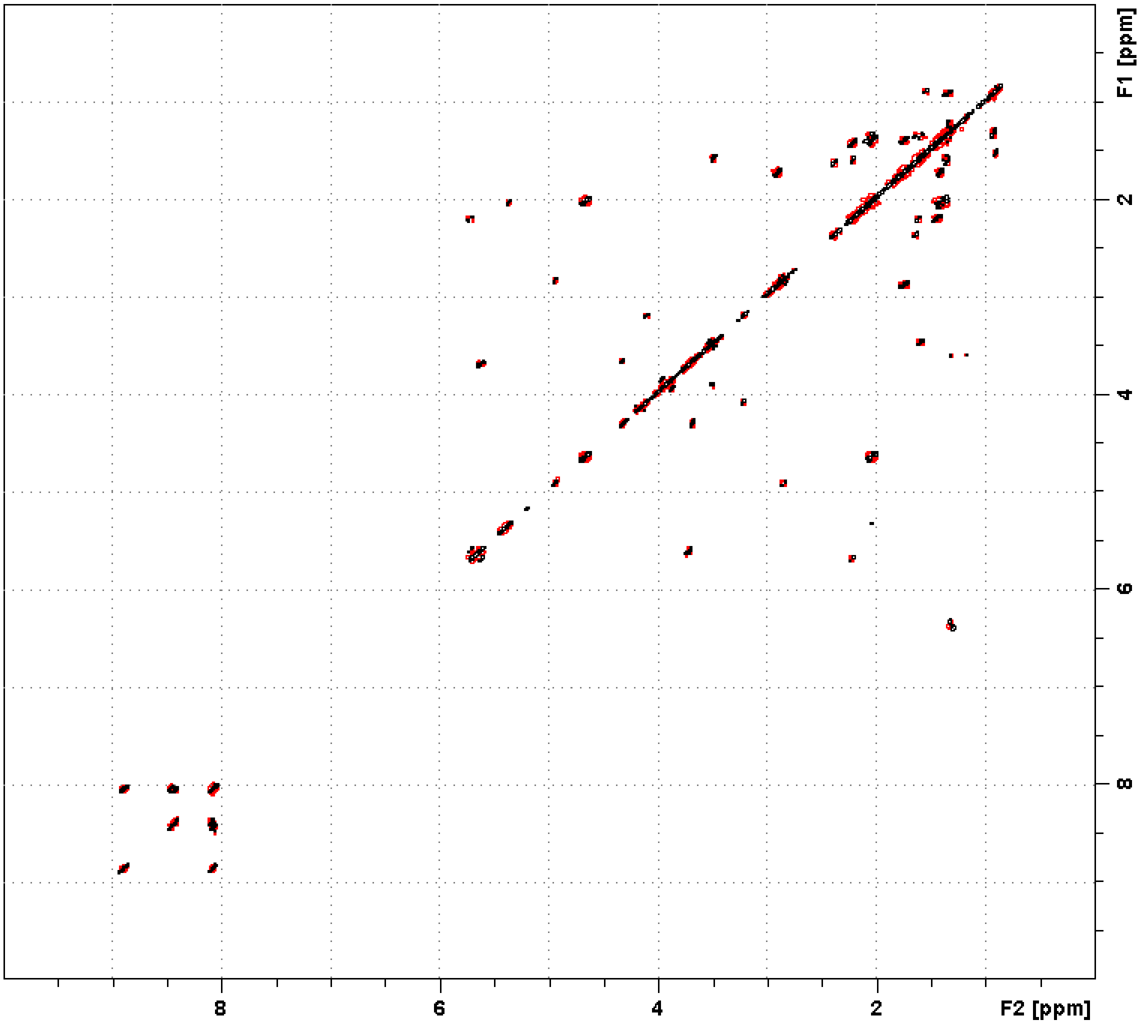

Figure S7. ^{1}H-^{13}C HSQC spectrum of compound **1**

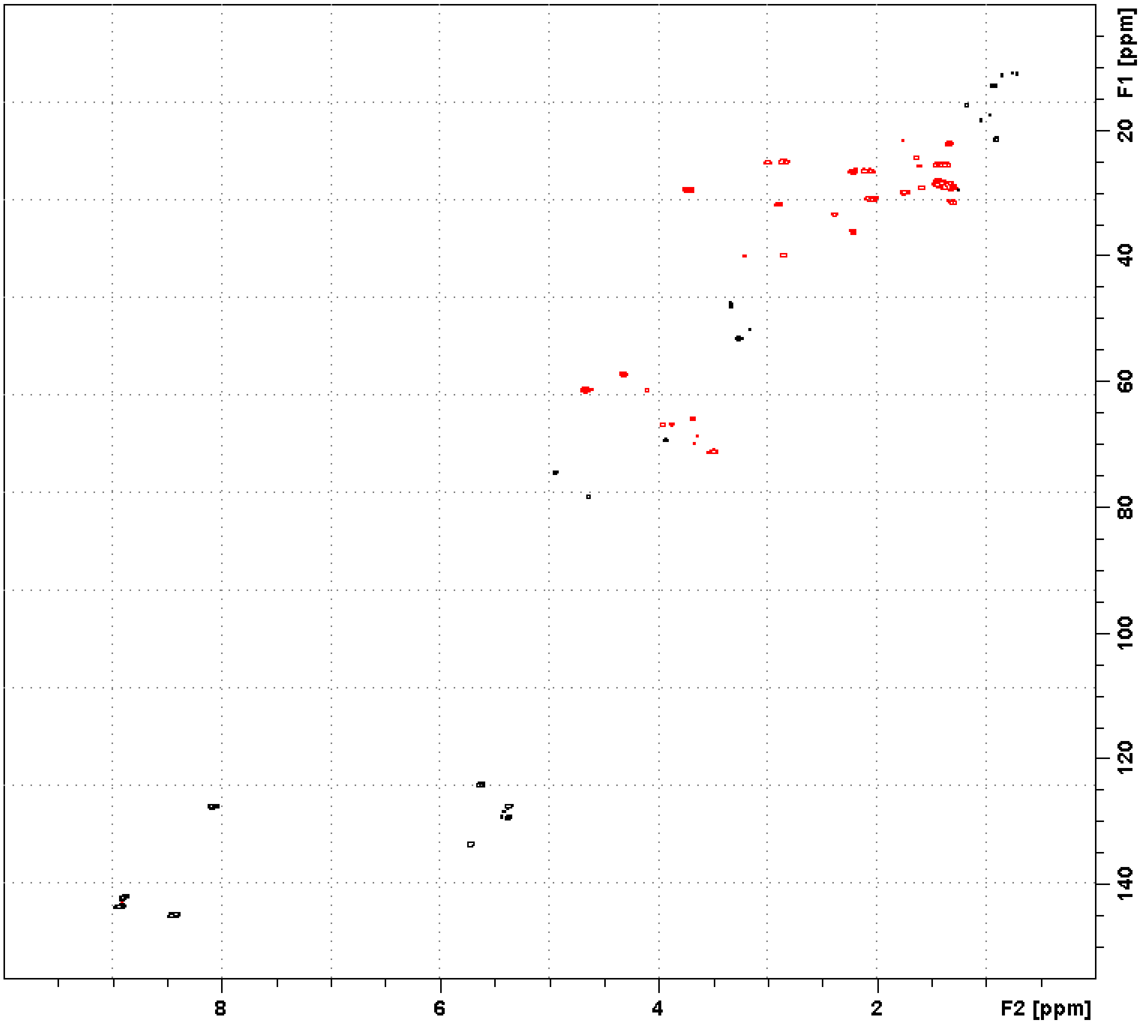

Multiplicity edited ^{1}H-^{13}C HSQC spectrum of **1** in methanol-d_4 at 298K shows methyl and methine resonances in black and methylene resonances in red. The peaks near 5.50,130.0 ppm could not be correlated to those of **1** and are assumed to be due to impurities.

Figure S8. ^{1}H-^{13}C H2BC spectrum of compound **1**

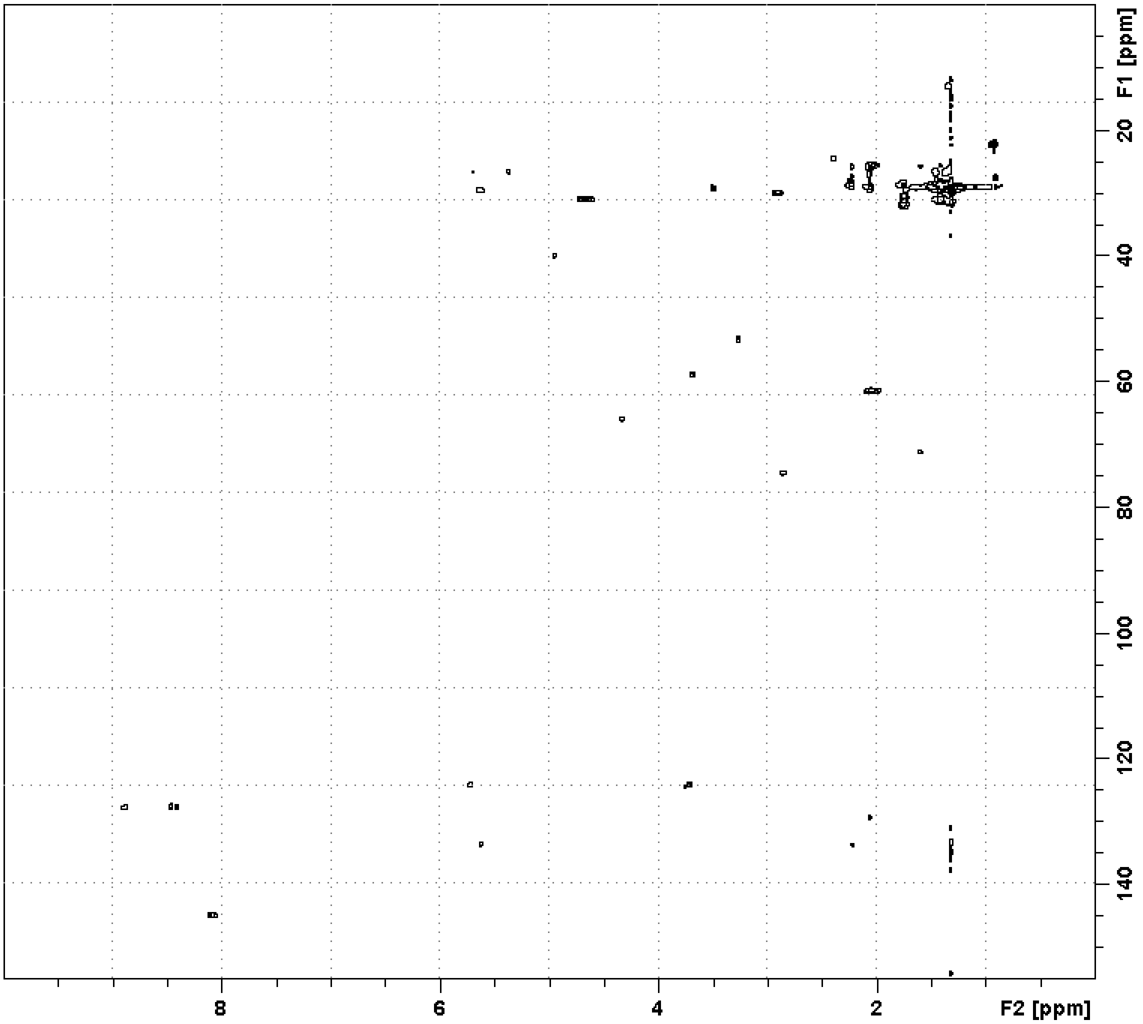

Figure S9. ^{1}H-^{13}C HMBC spectrum of compound **1**

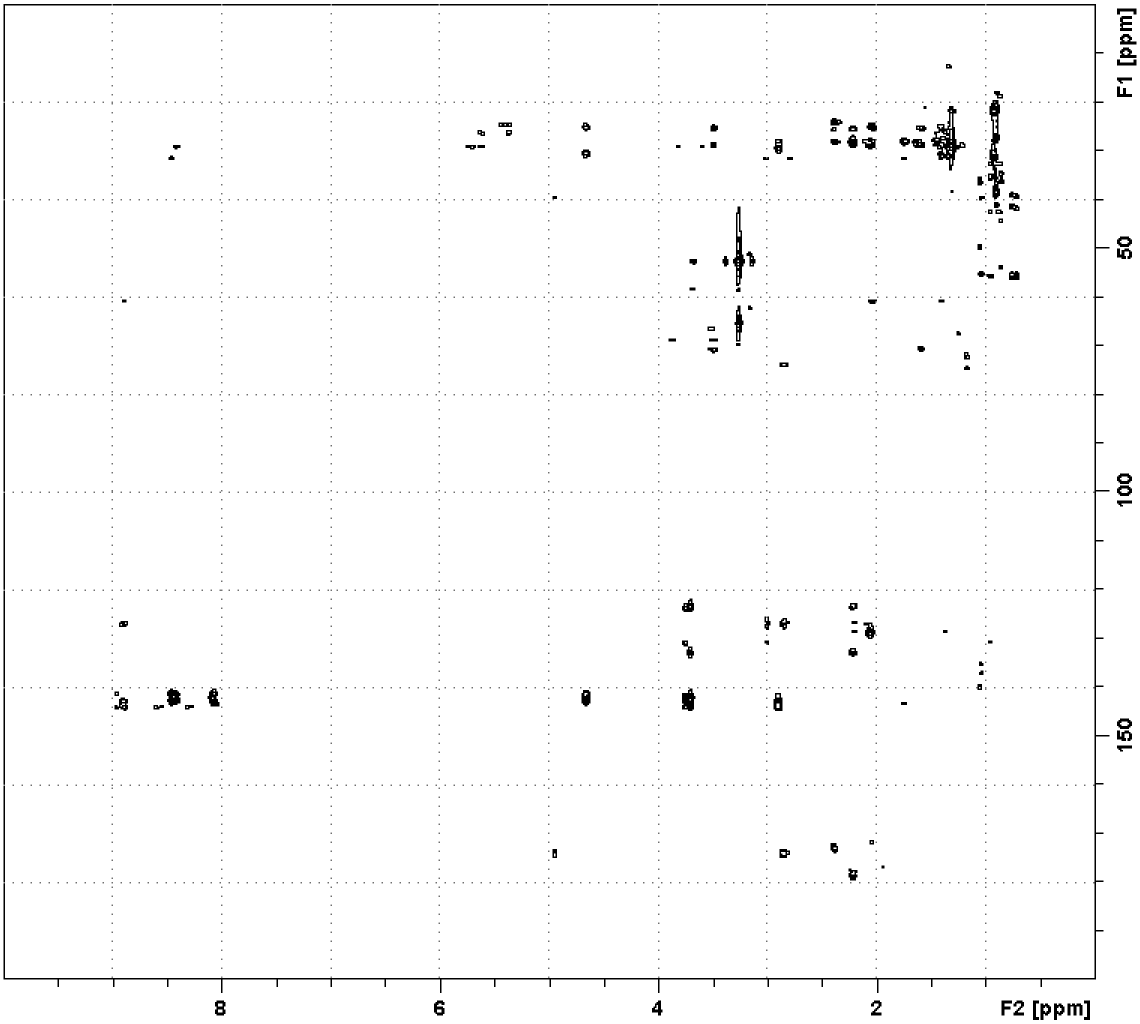

Figure S10. ^1H-^1H NOESY spectrum of compound **1**

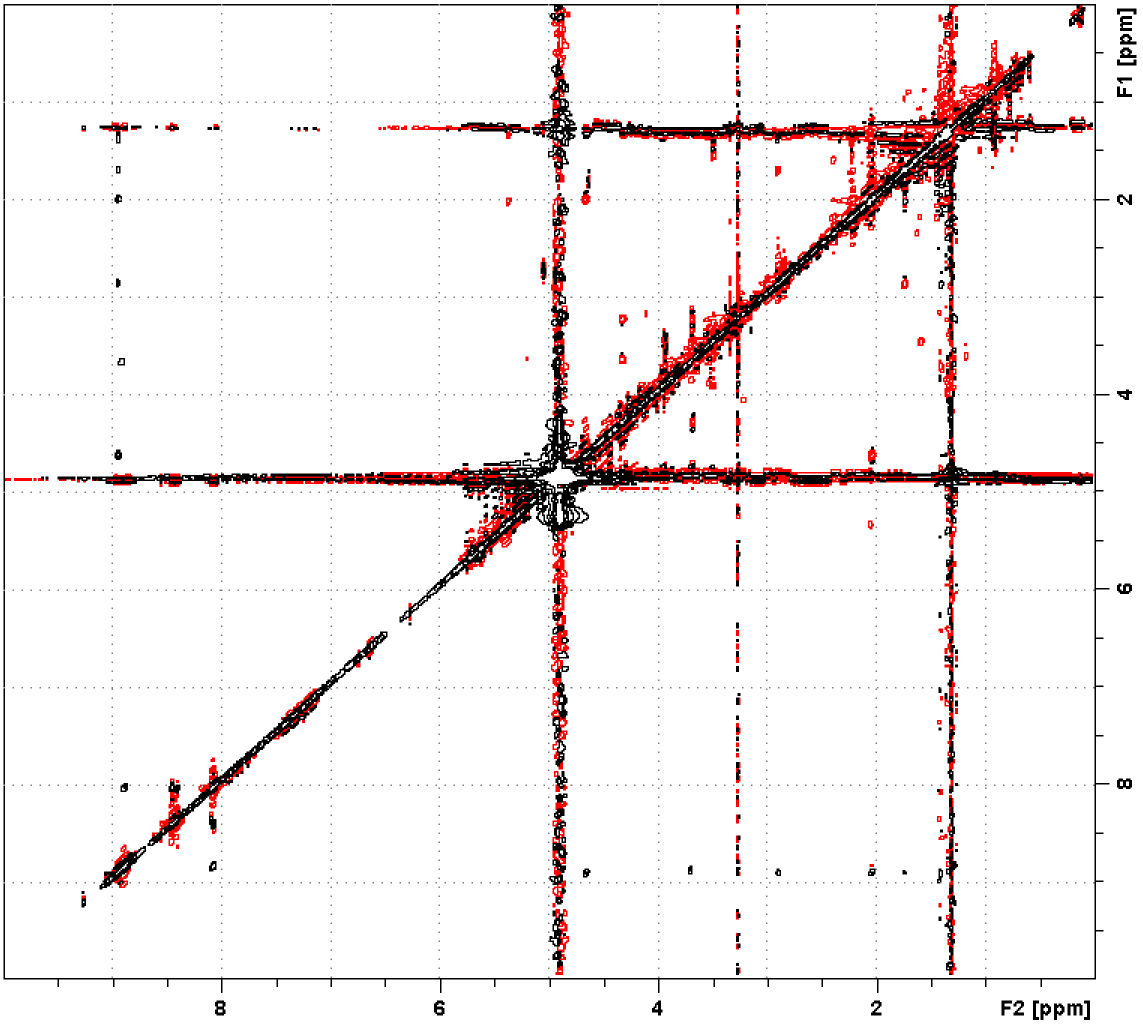

2D ^1H-^1H 500 ms NOESY NMR spectrum of compound **1** in methanol-d_4 at 600 MHz and 298 K. Black peaks indicate negative NOEs produced by slowly tumbling molecules and red peaks indicate positive NOEs produced by rapidly tumbling molecules. Note the NOE near the top left of the spectrum between aromatic H2 (8.95 ppm) and aliphatic H8 (2.04 ppm) is negative, while the NOE between aliphatics H8 (2.04 ppm) and H7 (4.64 ppm) is positive.

Figure S11. ^1H-^1H ROESY spectrum of compound **1**

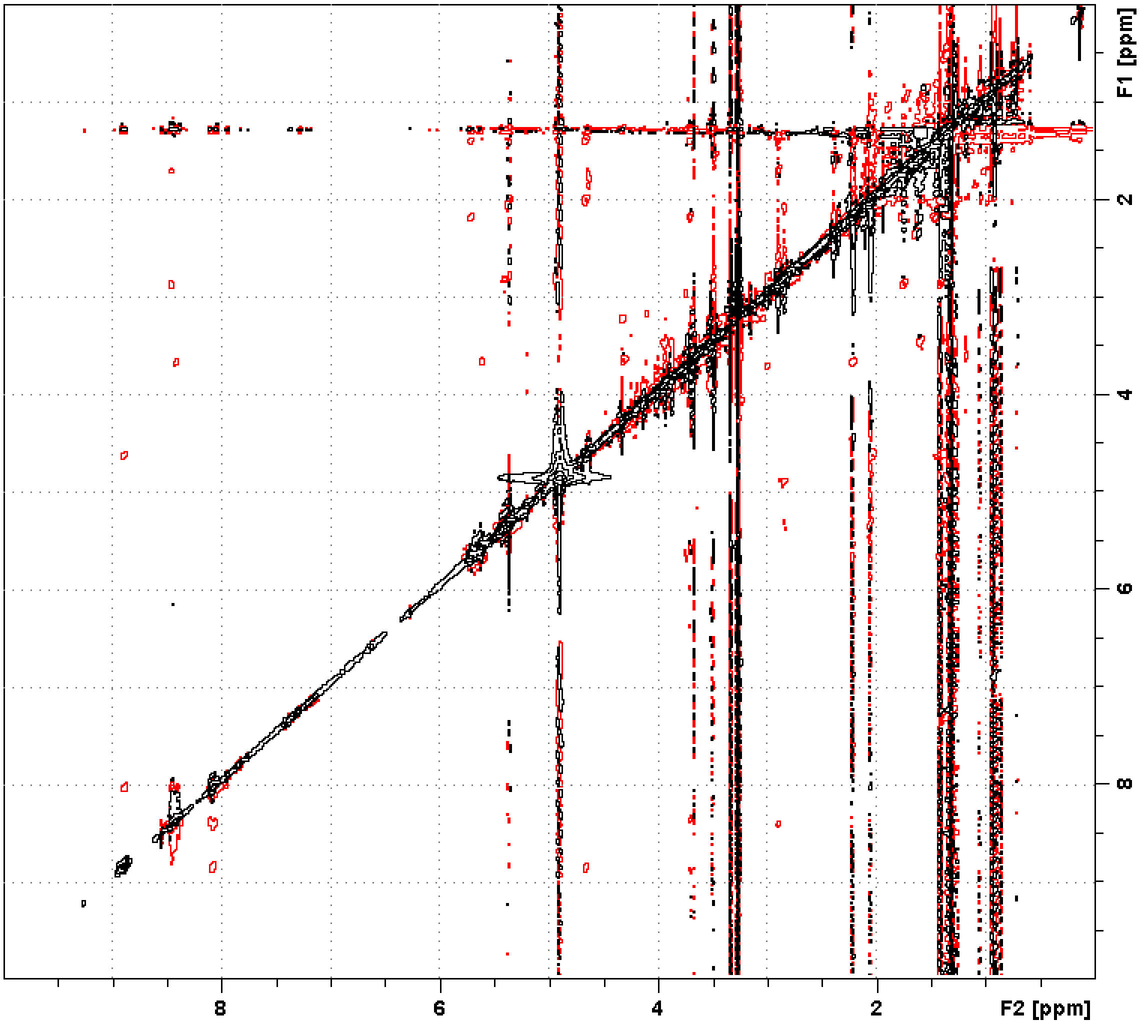

2D ^1H-^1H 300 ms ROESY NMR spectrum of compound **1** in methanol-d_4 at 600 MHz and 298 K. Red peaks indicate positive ROEs. Black peaks would identify resonances undergoing chemical exchange but none are present.
